# Development of an Automatic Testing Platform for Aviator’s Night Vision Goggle Honeycomb Defect Inspection

**DOI:** 10.3390/s17061403

**Published:** 2017-06-15

**Authors:** Bo-Lin Jian, Chao-Chung Peng

**Affiliations:** Department of Aeronautics and Astronautics, National Cheng Kung University, Tainan 70101, Taiwan; bo.lin.jian@gmail.com

**Keywords:** night vision goggles, military avionics systems, defect detection, auto focus, passive focusing

## Abstract

Due to the direct influence of night vision equipment availability on the safety of night-time aerial reconnaissance, maintenance needs to be carried out regularly. Unfortunately, some defects are not easy to observe or are not even detectable by human eyes. As a consequence, this study proposed a novel automatic defect detection system for aviator’s night vision imaging systems AN/AVS-6(V)1 and AN/AVS-6(V)2. An auto-focusing process consisting of a sharpness calculation and a gradient-based variable step search method is applied to achieve an automatic detection system for honeycomb defects. This work also developed a test platform for sharpness measurement. It demonstrates that the honeycomb defects can be precisely recognized and the number of the defects can also be determined automatically during the inspection. Most importantly, the proposed approach significantly reduces the time consumption, as well as human assessment error during the night vision goggle inspection procedures.

## 1. Introduction

Night vision goggles (NVGs) are used to enhance the visibility of helicopter crew members in low-light environments [1]. The basic NVGs structure is comprised of a mounting frame to hold all of the components, an objective lens to focus the night image onto the photocathode, a channel-plate proximity-focused image-intensifier, and a magnifying eyepiece with focusing adjustments to display the intensified image to the viewer [2]. The electro-optic system of the image intensifier detects and intensifies reflected energy in the visible range (400 to 700 nm) and the near-infrared range (700–1000 nm) of the electromagnetic spectrum [3]. The image quality of NVGs relies on the intensifier to amplify the detected electromagnetic signals [4]. The function of an image intensifier is to convert weak visible and near-infrared light to electrons, which are then converted into large quantities of secondary electrons via an electron amplifier (creating an electron cloud). These electrons collide with the screen to create visible light. The availability of NVGs directly influences mission safety during night-time reconnaissance such that regular maintenance is compulsory.

Image peculiarities commonly seen in NVGs include shading, edge glow, bright spots, dark spots, honeycomb, distortion, flicker, and scintillation [5]. Among these peculiarities, the honeycomb defect is also known as fixed pattern noise of a faint hexagonal form. A honeycomb-like pattern in the image is most often seen in high-light-level conditions [6]. If it is obvious or distracting, the image-intensifier should be replaced. Determining honeycomb defects are relatively more difficult than other types of defects because they lack a set reference and are much more difficult to identify with the naked eye. According to the current standard operation procedure, the aviator’s night vision imaging systems AN/AVS-6(V)1 and AN/AVS-6(V)2 remain reliant on manual calibration [7]. Prior to the calibration, technicians are going to be asked to enter a dark room for approximately twenty minutes to increase their sensitivity to faint light in darkness. This operation procedure definitely prolongs the total calibration time. Moreover, the goggles must be placed on a testing platform and technicians are required to perform calibration through the goggle’s eyepiece while manually adjusting the focus [8,9,10]. During the calibration, manual observation through the eyepiece of the NVGs and manual adjustment of focal length are performed simultaneously. Long inspection times induce negative physiological effects easily, such as loss of concentration and biases, which lead to improper calibration results.

To reduce the technical training time and to achieve efficient inspection of the NVGs, a camera was installed on the testing platform which captured the image through the NVGs. An auto-focusing algorithm and custom-design hardware were integrated to develop an automated defect detection system for NVGs. Automatic detection of honeycomb defects using image pattern recognition was proposed to reduce the calibration difficulty of manual operations.

Currently, there are two methods for auto-focusing, namely, active and passive auto-focusing. In active auto-focusing, add-on infrared [11] or other measuring tools [12] are used to measure the distance between the camera lens and target object. Passive auto-focusing involves calculating sharpness from single images obtained by the camera. Sharpness curves are then generated after calculating the sharpness of multiple images, where the peak values from the sharpness curve correspond to the optimal focal length [13,14,15]. This study adjusted focal length using the images obtained from the lens of the NVGs. Hence, a passive auto-focusing method was used. However, the key to this method is whether a correct in-focus point can be effectively calculated from the image information. The light source luminosity is also an important factor affecting the passive auto-focus system [16,17,18]. The ANVTP (TS-3895A/UV) used in this study provided a stable low-light environment, minimizing the effects from the light source. 

The optimal sharpness calculation methods can be categorized as the typical depth from focus (DFF) or depth from defocus (DFD) methods. DFD is commonly used in depth estimation and scene reconstruction. This method has relatively few calculation samples, which improves servomotor efficiency at the expense of accuracy degradation [19]. Due to noise in the image, stability, and precision need to be considered simultaneously. This study followed the similar sharpness calculations proposed by Pertuz et al. [20].

Based on the preceding introduction, this work dedicates to pursue efficient and precise automatic defect detection for NVGs. The main features include:

1. A novel searching algorithm, which is able to achieve fast and accurate focusing, is proposed. The main advantage of the developed method is also addressed through a comparison study.
2. Different sharpness estimation methods for NVGs are also considered for comparison studies.
3. A honeycomb defect detection process is proposed to automatic point out the number of defects and it corresponding locations. Therefore, the detection procedure can be realized efficiently and objectively.

## 2. Proposed Approach

This study employed an aviator’s night vision testing platform (TS-3895A/UV) to provide the required low-light environment during calibration and inspection [21]. To achieve auto defect detection purpose, the main procedure includes two parts. Firstly, fast auto focusing, which provides clear image for honeycomb detection, is addressed. Secondly, an image processing algorithm is proposed to detect the honeycomb defect. Detail experimental results are discussed in Section 2. In Section 2.1, hardware specifications are provided. Performances conducted by different sharpness measurement methods are given in Section 2.2. The proposed method and its efficiency is addressed in Section 2.3. Finally, the detail honeycomb detect detection procedure is introduced in Section 2.4. 

### 2.1. A. Experimental Setup

**Figure 1:**
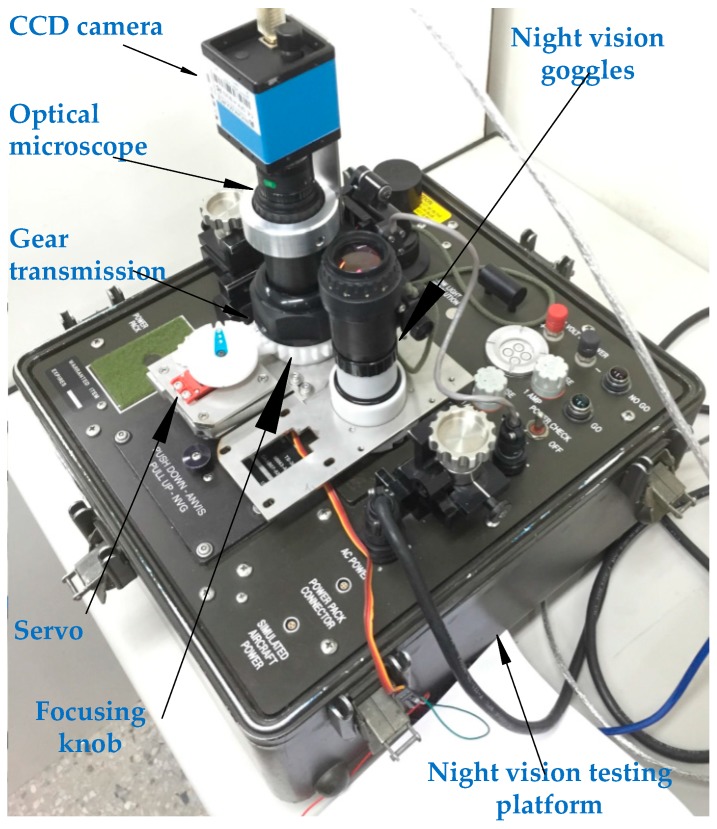
System installation.

An optical microscope (Computer M0814-MP2, CBC Americas, Cary, NC, USA) with CCD (DFK 41BU02, Imaging Source, Charlotte, NC, USA) detection was used in this study. The CCD camera had a size of 50.6 mm × 50.6 mm × 50 mm and weight of 265 g. The image dimensions were 1280 × 960 pixels with a pixel size of 18.6 μm and a focal length of 8 mm. A DC servo driver (S03Q3, Grand Wing Servo-Tech Co., Ltd., Upland, NY, USA) was used to rotate the focusing knob of the NVGs. The servo controller was implemented using an Arduino Mega 2560 board (Smart Projects, Olivetti, Italy), which generates pulse width modulation (PWM) signals of 0.9–2.1 ms for ±60° movement range of the servomotor. The resulting system is shown in Figure 1, where a fixed mechanism is used to fix the camera on the NVGs and a servo transmission mechanism is implemented to adjust the focal length of the NVGs.

### 2.2. Process for Passive Auto-Focusing

**Figure 2:**
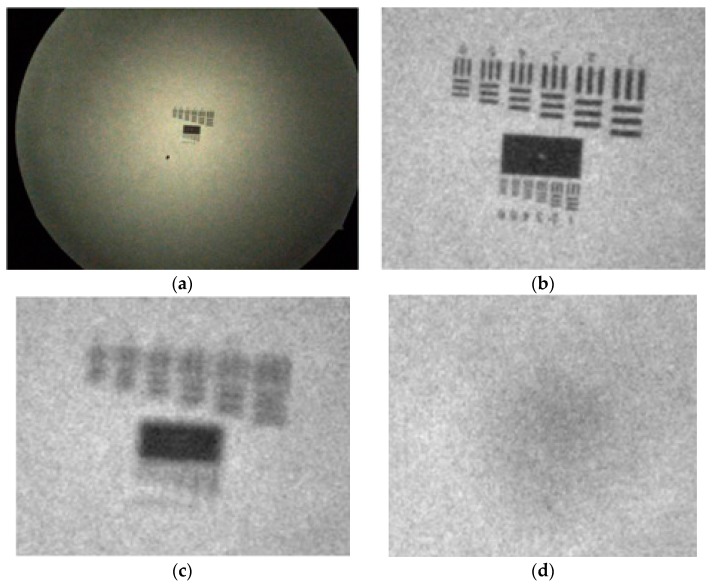
Image preprocessing. (**a**) Initial image; (**b**) Sharp image (correctly focused); (**c**) Blurry image (slightly out of focus); (**d**) Blurry image (severely out of focus).

Passive auto-focusing involves sharpness measure from images obtained by the camera. Sharpness curve generated according to image sharpness of different focal length is a good indication of sensitivity to in-focal status [13,14]. Before establishing a comprehensive process for passive auto-focusing in this study, the classic sharpness measure reviewed by Pertuz et al. was studied [20]. For image sharpness calculation, twenty-eight methods are considered, which can be divided into six categories including, gradient-based operators, Laplacian-based operators, wavelet-based operators, statistics-based operators, DCT-based operators, and miscellaneous operators. To verify the feasibility, the experiments were conducted through the same image capturing device, test target as well as light source. As shown in Figure 2, the target images Figure 2b–d is 200 × 250 pixel images obtained from the color image Figure 2a, of size 960 × 1280 × 3 pixels through the NVGs. The sharpness curves under the 28 method were then estimated for the target images.

**Figure 3:**
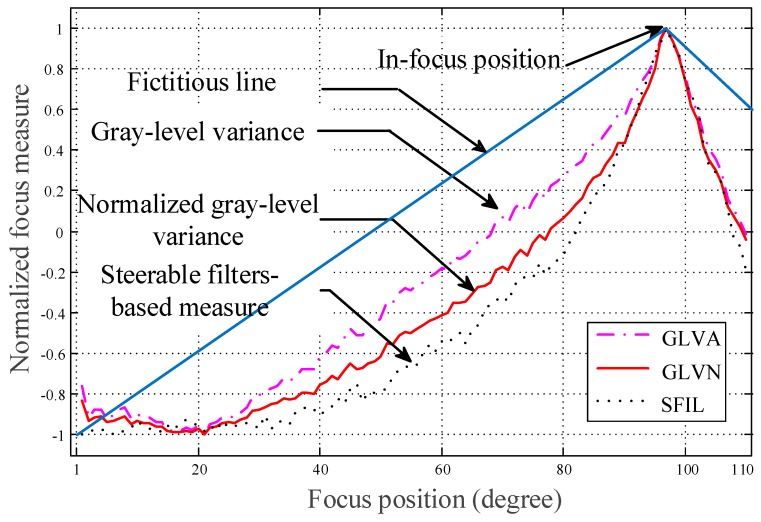
Sharpness to servomotor rotation angle.

By the considerations of accuracy, computation time, correlation coefficients to the optimal value and entropy as shown in Table 1, the normalized gray-level variance sharpness measure will be the best candidate and is, thus, applied in this study. In Figure 3, a fictitious line is assumed as a sharpness reference. Based on the evaluation of entropy, gray-level variance is with lower performance. On the contrary, steerable filters-based measure is with better performance on entropy. However, referring to Table 1, the normalized gray-level variance measure is able to achieve the best average elapsed time, correlation coefficient and entropy. The normalized gray-level variance measure [20] is described as follows:

(1) $$FM=\frac{1}{\bar{I}\left(M-1\right)\left(N-1\right)}\sum_{x=1}^{M}\sum_{y=1}^{N}{\left(I\left(x,y\right)-\bar{I}\right)}^{2}$$

where I(x,y) denotes the pixel intensities at the position (x,y) and $\bar{I}$ is the average intensity of the image which is of size M×N.

**Figure 4:**
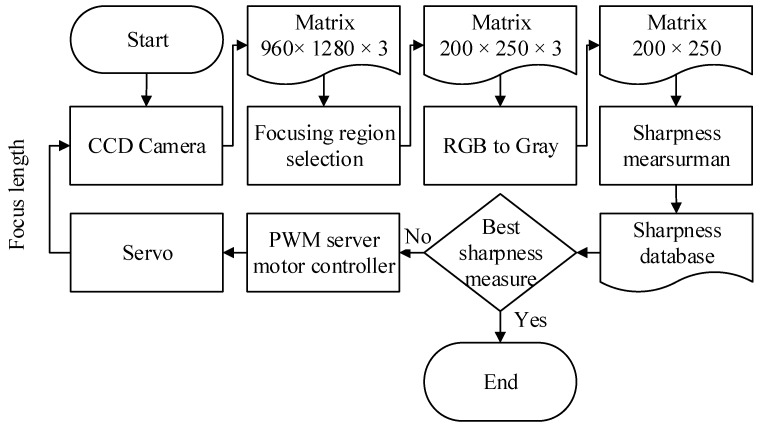
Passive auto-focusing process for the NVGs.

The required focus length of the NVGs on the testing platform is adjusted by a servomotor driven by a microprocessor Arduino Mega 2560 board. The focal length can then be tuned such that the best sharpness is obtained. The complete passive auto-focusing process is described in Figure 4. Firstly, a single image is captured from a CCD camera, where a region of interest is selected with the size of 200 × 250 × 3 pixels. The interested image is transformed in to a gray one and then the corresponding sharpness is calculated. Once the best sharpness is attained, the procedure is finished. Otherwise, the focus length is going to be further modified via a servo motor for the next iteration.

### 2.3. Search Approaches

Since the peak point is the optimal focal point, the searching aim is going to find an effective approach to estimate the optimal solution. Due to excessive time requirements and ineffective global search strategy, peak search strategies have been successively proposed [22,23]. A hill-climbing search (HCS) [24] and rule-based algorithm are both popular methods, which have been widely applied for peak point searching [25].

Moreover, this paper proposed a gradient-based variable step search method. The best progression step size is estimated by evaluating the variation of the normalized gray-level, which can be formulated as follows:

(2) $$\theta_{Opt}^{i+1}=\theta_{Opt}^{i}+\lambda \frac{\left(\theta_{Opt}^{i}-\theta_{Opt}^{i-1}\right)}{FM(\theta_{Opt}^{i})-FM(\theta_{Opt}^{i-1})}$$

where $\theta_{Opt}^{i}$ denotes as the best angle position, i is the progression steps of servo motor, and λ is a scaling factor. The normalized gray-level variance under the $\theta_{Opt}^{i}$ is returned by $FM(\theta_{Opt}^{i})$. 

In order to improve the efficiency of autofocus, different searching methods were applied for comparison purposes. Firstly, the focus measurement from different rotation angles is collected, where the focus measurement is evaluated by Equation (1). This step is used to construct a truth database for the following comparisons. Secondly, using five search algorithms, including global, hill-climbing, binary, rule-based, and gradient-based variable step search methods, to calculate the iterations and estimate accuracy. The results are summarized in Table 2. The hill-climbing search method is not able to find the optimal solution. The accuracy by using binary-search method is less than the one obtained by applying gradient-based variable step search method. For rule-based search method, the number of iterations are higher than other search methods. With the consideration of a trade-off between iterations and accuracy, it can be concluded that the gradient-based variable step search method will be the best candidate. 

### 2.4. Honeycomb Defect Detection Procedure

**Figure 5:**
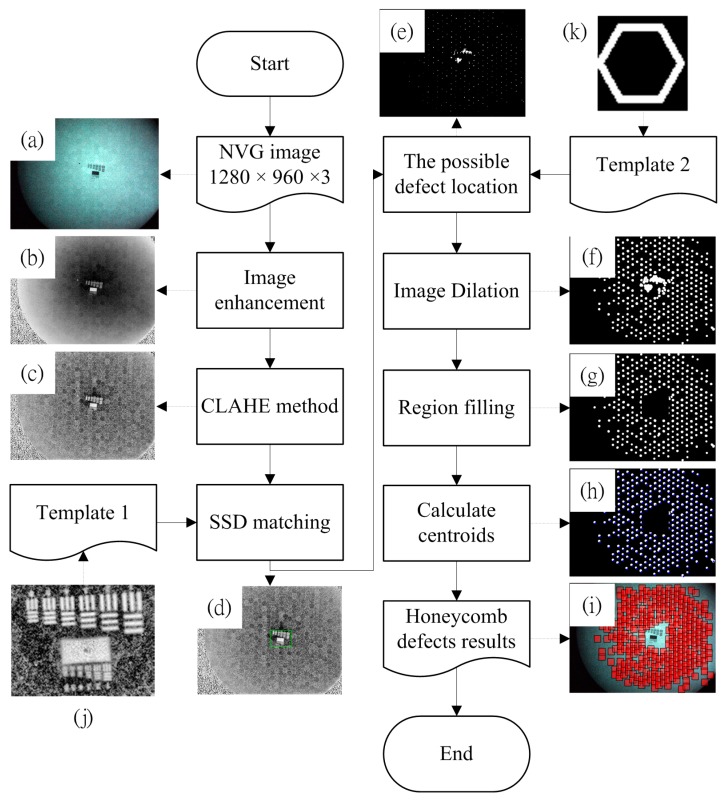
(**a**–**k**) Automatic honeycomb defect detection process.

After completing the process as given in Section B, automatic honeycomb defect detection can be performed as the schematic diagram shown in Figure 5. The step-by-step procedure is described as follows:

1. A color image of dimensions 960 × 1280 × 3 pixels is obtained via a CCD camera, as shown in Figure 5a.
2. Since the green color array component from the RGB color system has the most obvious honeycomb defects and the blue color array component has the least, the green array is subtracted from the blue array after extracting them from the color image to reduce processing dimensions. This allows a reduction in the non-honeycomb defect region components and accentuates the honeycomb defect region information. The obtained gray level image is shown in Figure 5b.
3. The contrast limited adaptive histogram equalization (CLAHE) method [26] is introduced to process the correlation between the pixel locations and the other gray level pixels. An increased contrast ratio is achieved and the defect regions are accentuated, as shown in Figure 5c.
4. The sum square difference (SSD) method [27] is used to detect the positions of the mark, which is applied to the focusing process, using the template image shown in Figure 5j. The mark area is highlighted by a rectangular region shown in Figure 5d. The matching measure SSD is defined as follows:
  (3) $$SSD(x,y)=\sum_{m=0}^{M}\sum_{n=0}^{N}{\left(R\left(x+m,y+n\right)-S\left(m,n\right)\right)}^{2}$$
  where R(x+m,y+n) is the search region (or the so-called primary image) at the position (x+m,y+n) and S(m,n) is template image at the position (m,n) which is of size M×N.
5. To find the location of the honeycomb defects, the binary template, Figure 5k, of size 61 × 58 pixels is introduced to carry out a “shift” operation for the image obtained in step 3. During the operation, a sub-image will be binarized using a threshold of its average intensity. If the amount of the corresponding pixels having the same value with “template 2” is greater than 55% of the total amount, the sub-image will set to be white. Otherwise, it will be black. The “white” area indicates possible location of the honeycomb defect as shown in Figure 5e. Hence, this step will produce a binary image, wherein the white points mark locations that are similar to honeycomb defects. The process of calculated possible defect location image is summarized in Table 3.
6. White color expansion is performed on the resulting binarized image of step 5 to group the honeycomb defects around the neighborhood, as shown in Figure 5f.
7. The region of mark identified in step 4 is removed for further processing. After the removal, the image becomes Figure 5g.
8. For honeycomb defect positioning, the centroid of each white-colored dot indicates the upper left corner of a honeycomb defect. The result is shown in Figure 5h.
9. The honeycomb defect region is superimposed on top of image Figure 5a, as shown in Figure 5i.

Improvement percentage: $\frac{\left(\mathrm{Iterations Global}-\mathrm{Iterations Other}\right)}{\mathrm{Iterations Global}}\times 100\%$; Rule-based search parameter (Initial, Coarse, Mid, Fine); Gradient-based variable step search parameter (λ, Initial $\theta_{Opt}^{i}$, Initial $\theta_{Opt}^{i-1}$); The iteration stop condition is: $\left|\Delta \theta_{Opt}\right|:=\left|\theta_{Opt}^{i}-\theta_{Opt}^{i-1}\right|<\theta_{servo}$, where $\theta_{servo}$ is the minimal step size of the servo.

## 3. Results and Discussion

The results from Table 2 shows that the global search is able to find the correction peak point. However, it causes 110 times iteration and thereby this method is time consuming. For the hill-climbing search, it is sensitive to sharpness curve and thus the searching robustness is not high enough. For the binary search, it leads to successful results. Nevertheless, only sub-optimal solution is available. For the rule-based search, the number of iterations are less than those by global-search but greater than those by the binary search. This method is able to find the correct solution under that the initial parameter is 12, coarse parameter is 3, mid-parameter is 2, and the fine parameter is 1. However, determination of parameters is highly dependent on technicians’ experiences. For detailed parameter definitions please refer to [25]. 

In this study we developed a gradient-based variable step search method. By setting λ and an initial position $\theta_{Opt}^{i}$ to be 0.28° and 60°, respectively, the correct peak point can be latched. Moreover, the number of iterations is less than those by using rule-based search. Synthesizing all of the experiences, Table 2 shows that the best estimation by the proposed gradient-based search is better than the best one obtained by the rule-based approach, and a high accuracy (i.e., L1) can be guaranteed as well.

The experiment for defect detection is carried out by using 30 NVG samples, which are pre-identified by well-trained technicians. Among the test samples, fifteen of them are with honeycomb defects and the others are defect-free. The gradient-based peak search method (2) is applied in this approach. The normalized gray-level variance sharpness measure is carried out on Windows 8.1 64-bit system running under an Intel Core i7 3770 CPU with 8 GB memory. The system run-time for auto-focusing is less than 15 s, which includes the time required for image capture, sharpness calculation and servomotor control.

**Figure 6:**
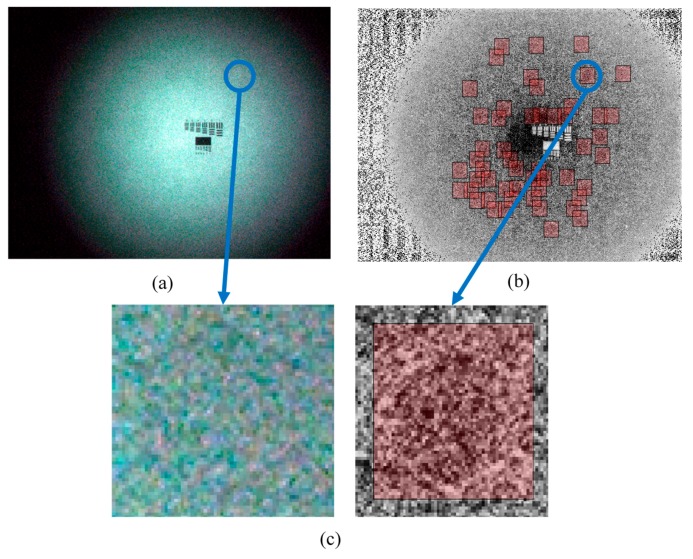
Image processing results for slight defect of honeycomb. (**a**) The slight defect of honeycomb. (**b**) The slight defect of honeycomb’s result show at CLAJE image. (**c**) Zoom-in of the slight honeycomb defect.

The detail inspection results of the 30 NVG samples by using the proposed approach are listed in Table 4. The results illustrate that the honeycomb defect detection system is able to identify defective and non-defective NVGs separately. Especially for the blurred honeycomb defect as shown in Figure 6a–c, it will be very difficult to identify even by professional technicians. However, Figure 6a–c evidently show that this issue can be solved with the aid of the proposed method. Using the same hardware configuration for auto-focusing, the entire image processing time for defect identification was within 45 s via the use of MATLAB^®^ Version 2016a (The Mathworks, Natick, MA, USA) parallel computing toolbox. The algorithm can be further implemented by C++ to reduce the processing time down to 5 s. For the current manual procedure, it takes at least 30 min. Therefore, experiments evidently verify that the developed hardware configurations and algorithms can achieve precise and effective inspections.

## 4. Conclusions

This paper is concerned with the inspection of the aviator’s night vision imaging system AN/AVS-6(V)1 and AN/AVS-6(V)2. Twenty-eight types of sharpness measures were investigated and, finally, a gradient-based variable step searching was used to achieve fast auto-focusing system. Experimental results indicate that using the normalized gray-level variance operator together with the gradient-based peak search method ensures both the efficiency and accuracy of the inspection. Moreover, an automatic honeycomb defect detection algorithm was proposed to address the defective level of the night vision instrument and was further verified by experiments. By using the proposed approach, the inspection time for manual calibration can be significantly attenuated. Meanwhile, the variance due to personal subjective understanding regarding in-focus, as well as other human errors, could be avoided. Therefore, the high maintenance quality for NVGs is going to be guaranteed through a systematic, scientific, and objective way.

## Figures and Tables

**Table 1:** Statistical sharpness estimation comparison.

	In-Focus Result	Average Elapsed Time (s)	Correlation Coefficient	Entropy
Criterion	=Y	<0.01	>0.2	<0.4
Absolute Central Moment	N	0.0009	−0.2280	0.5203
Brenner’s focus measure	Y	0.0012	−0.1168	0.5942
Image contrast	Y	9.0607	0.0420	1.1801
Image curvature measure	Y	0.0049	0.0980	2.9132
DCT energy ratio	Y	5.2191	0.1596	0.5203
DCT reduced energy ratio	Y	5.0998	0.1667	2.2880
Gaussian derivative	Y	0.0026	0.2570	0.7414
Gray-level variance	Y	0.0008	0.3519	0.5203
Gray-level local variance	Y	0.0030	0.1500	0.7414
Normalized gray-level variance	Y	0.0007	0.3330	0.2980
Gradient energy	Y	0.0010	0.0162	4.6710
Thresholded absolute gradient	Y	0.0013	−0.0181	3.1345
Squared gradient	Y	0.0006	0.0100	4.7005
Helmli and Scherer’s mean	Y	0.0024	0.0800	4.0896
Histogram entropy	N	0.0006	0.3667	2.6489
Histogram range	N	0.0004	0.3840	3.4788
Energy of laplacian	Y	0.0013	−0.1166	1.8104
Modified Laplacian	Y	0.0016	−0.1074	0.7414
Variance of Laplacian	Y	0.0014	−0.0882	0.4463
Diagonal laplacian	Y	0.0026	−0.0896	0.2980
Steerable filters-based measure	Y	0.0067	0.3288	0.4463
Spatial frequency measure	Y	0.0013	0.0164	5.5735
Tenengrad	Y	0.0019	0.0901	4.8222
Tenengrad variance	Y	0.0020	0.0706	0.2980
Vollath’s autocorrelation	Y	0.0007	0.1072	0.5203
Sum of wavelet coefficients	N	0.0097	−0.2256	0.5942
Variance of wavelet coefficients	Y	0.0087	−0.15375	1.1801
Ratio of wavelet coefficients	Y	0.0210	−0.0285	2.9132

SSRA: sharpness to servo rotation angle data; In-focus result: Y → success, N → fail; Average elapsed time: SSRA computation time cost; Correlation coefficient: correlation coefficient calculation between the fictitious line (as shown in Figure 3) and SSRA; Entropy: uncertainty evaluation of SSRA; A higher correlation coefficient implies the result tends to meet expectation. A higher entropy means the sharpness curve is with higher uncertainty and thus the precision of the in-forces position will be relatively low.

**Table 2:** Comparison of studied search algorithms.

	Iterations	Accuracy (L1: ±0, L2: ±1, L3: ±2, Failure)	Improvement Percentage (%)
Global search	110	L1	N/A
Hill-climbing Search	2	failure	failure
Binary search	8	L2	92.73
Rule-based search (12-3-2-1)	39	L1	64.55
Rule-based search (12-4-3-2)	24	L2	78.18
Rule-based search (12-5-4-3)	19	L2	82.73
Rule-based search (12-6-5-4)	16	L3	85.45
Gradient-based variable step search (0.25, 60, 1)	9	L2	91.82
Gradient-based variable step search (0.26, 60, 1)	11	L2	90
Gradient-based variable step search (0.27, 60, 1)	8	L2	92.73
Gradient-based variable step search (0.28, 60, 1)	14	L1	87.27
Gradient-based variable step search (0.29, 60, 1)	8	L2	92.73
Gradient-based variable step search (0.30, 60, 1)	9	L2	91.82

**Table 3:** The process for defect location detection.

Inputs: Figure 5c,k. Outputs: The possible defect location image. Calculate the average intensity of MaskArea image: $$AvgGray=\frac{1}{M\times N}\sum_{x=1}^{M}\sum_{y=1}^{N}MaskArea\left(x,y\right)$$ M is width of an image, N is height of an image, MaskArea is obtained by a shift operation. By step1, let AvgGray be the threshold of MaskBW binarization image: $$\begin{array}{l} \left(AvgGray\geq MaskArea\left(x,y\right)\to MaskBW\left(x,y\right)=1\right)\\ \&\left(\text{\textasciitilde{}}AvgGray\geq MaskArea\left(x,y\right)\to MaskBW\left(x,y\right)=0\right) \end{array}$$ The MaskBW and Figure 5k ($Temp_{\theta }\left(x,y\right)$) image that we calculate the area of same pixels, that we can get CountMat matrix (θ is template rotated from 1 to 60 degree): $$\begin{array}{l} \left(MaskBW\left(x,y\right)=Temp_{\theta }\left(x,y\right)\to CountMat\left(x,y\right)=1\right)\\ \&\left(\text{\textasciitilde{}}MaskBW\left(x,y\right)=Temp_{\theta }\left(x,y\right)\to CountMat\left(x,y\right)=0\right) \end{array}$$ Sum the value in CountMat matrix. $$Count=\sum_{x=1}^{M}\sum_{y=1}^{N}CountMat\left(x,y\right)$$ Using binarization image (PossibleDefect) shown that could be the area of defective honeycomb. Threshold is the amount of the corresponding pixels having the same value with Temp(x,y) is 55% of the total amount: $$\begin{array}{l} \left(Count\geq Threshold\to PossibleDefect\left(x,y\right)=1\right)\\ \&\left(\text{\textasciitilde{}}Count\geq Threshold\to PossibleDefect\left(x,y\right)=0\right) \end{array}$$

**Table 4:** Experiment results for honeycomb defect detection.

Sample Condition (Inspected by Experts)	Sample Item	Amount of Honeycomb Defects Detected by the Algorithm
Honeycomb defect	1	1
	2	5
	3	2
	4	46
	5	2
	6	5
	7	27
	8	57
	9	14
	10	245
	11	7
	12	4
	13	8
	14	27
	15	11
Non-honeycomb defect	1	0
	2	0
	3	0
	4	0
	5	0
	6	0
	7	0
	8	0
	9	0
	10	0
	11	0
	12	0
	13	0
	14	0
	15	0
